# Localization and Edge-Based Segmentation of Lumbar Spine Vertebrae to Identify the Deformities Using Deep Learning Models

**DOI:** 10.3390/s22041547

**Published:** 2022-02-17

**Authors:** Malaika Mushtaq, Muhammad Usman Akram, Norah Saleh Alghamdi, Joddat Fatima, Rao Farhat Masood

**Affiliations:** 1 Department of Computer and Software Engineering, National University of Sciences and Technology, Islamabad 44000, Pakistan; mmushtaq.ce19ceme@ce.ceme.edu.pk (M.M.); usman.akram@ceme.nust.edu.pk (M.U.A.); 2 Department of Computer Sciences, College of Computer and Information Sciences, Princess Nourah Bint Abdulrahman University, P.O. Box 84428, Riyadh 11671, Saudi Arabia; 3 Department of Computer Science, Bahria University, Islamabad 44000, Pakistan; joddat.fatima@bahria.edu.pk; 4 Department of Electrical Engineering, Capital University of Science and Technology, Islamabad 44000, Pakistan; farhatmasood.fm@gmail.com

**Keywords:** deep learning, localization, lumbar lordortic angle, lumbosacral angle, lumbar spine, edge-based segmentation

## Abstract

The lumbar spine plays a very important role in our load transfer and mobility. Vertebrae localization and segmentation are useful in detecting spinal deformities and fractures. Understanding of automated medical imagery is of main importance to help doctors in handling the time-consuming manual or semi-manual diagnosis. Our paper presents the methods that will help clinicians to grade the severity of the disease with confidence, as the current manual diagnosis by different doctors has dissimilarity and variations in the analysis of diseases. In this paper we discuss the lumbar spine localization and segmentation which help for the analysis of lumbar spine deformities. The lumber spine is localized using YOLOv5 which is the fifth variant of the YOLO family. It is the fastest and the lightest object detector. Mean average precision (mAP) of 0.975 is achieved by YOLOv5. To diagnose the lumbar lordosis, we correlated the angles with region area that is computed from the YOLOv5 centroids and obtained 74.5% accuracy. Cropped images from YOLOv5 bounding boxes are passed through HED U-Net, which is a combination of segmentation and edge detection frameworks, to obtain the segmented vertebrae and its edges. Lumbar lordortic angles (LLAs) and lumbosacral angles (LSAs) are found after detecting the corners of vertebrae using a Harris corner detector with very small mean errors of 0.29° and 0.38°, respectively. This paper compares the different object detectors used to localize the vertebrae, the results of two methods used to diagnose the lumbar deformity, and the results with other researchers.

## 1. Introduction

**Figure 1:**
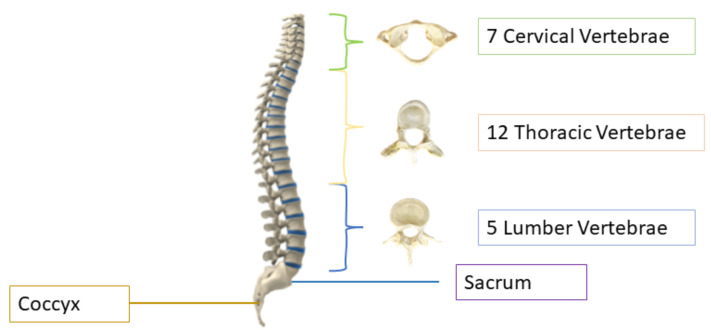
Structure of human spine [12].

Spine deformity can occur by birth, due to aging, injury, or due to spine surgery. Road accidents are the main cause of spinal injuries due to increasing rate of auto and motor vehicles. In 2013, the World Health Organization (WHO) presented key facts regarding spinal injuries and deformities showing that every year almost 250,000 to 500,000 people suffer from spine issues [1]. According to the 2016 American Journal of Public Health [2], after stroke, spine issues are the second leading cause of paralysis. The human spine consists of 26 vertebrae; the first seven in the neck are called cervical, the twelve in the torso are called thoracic, and the five in the lower back are called lumbar vertebrae, as shown in Figure 1. The other two are sacrum and coccyx. The area of the low back, also known as the lumbar region, starts below the rib cage [3]. The lumbar vertebrae, numbered from L1–L5, are the largest in size and are more prone to deformity because they are responsible for carrying the weight of the body [4]. About 80% of the population suffers lower back pain in their lives [5], and it is the third most common reason for doctor visits, costing Americans more than USD 50 billion each year [6]. Imaging tests can help the doctors in diagnosing the lumbar spine deformity and they can correlate it with pain symptoms. Diagnosing the deformity is a laborious task and clinicians require manual methods or computer-assisted diagnoses tools which act as a brain of doctors; they have improved the clinical identification, and are less prone to errors. Conventional manual diagnoses are prolonged and there can be variability in manual diagnoses [7,8]. Automated systems based on artificial intelligence (AI) can help to lessen the diagnostic errors caused by human clinical practice [9,10,11] and they can be used to assist the clinicians in diagnosing the spinal disorders.

Magnetic resonance imaging (MRI) and computed tomography (CT) technologies are used to detect various spinal disorders by machine learning (ML) techniques which assist the surgeons and physicians to diagnose the disease without using time-consuming manual methods. Timely diagnoses of spine deformities can prevent the patient from dangerous consequences and help in treating the disease at its early stage. MRI scan is good at detecting small herniation of discs, pressed nerves, and soft-tissue-related issues, while CT is more useful in detecting the moderate- and high-risk spinal fractures and injuries due to its clear bones’ structure [13].

AI has been very popular in medical imaging in the past few years, and it helps the clinicians and doctors to diagnose various diseases. As the number of imaging modalities are increasing, they support the clinicians to diagnose but they lack efficiency and accuracy; however, AI has changed the way people process large amounts of data [14]. Our objective is to develop a diagnostic system which is based on object detector framework and can be used to detect the lumbar spine deformities using machine learning tools. Manual labeling is outdated and automated methods can save the precious time of doctors.

The paper is organized as follows: Section 2 describes the related work about the models used for vertebrae identification and localization; Section 3 discusses the dataset used in our proposed technique and covers the detailed methodology, results are analyzed in Section 4 and the conclusion is presented in Section 5.

## 2. Related Work

AI is growing vastly in medical imaging, and automated systems have been developed by many researchers to diagnose different diseases and help the doctors to choose less invasive surgical procedures. Many studies have been carried out on lumbar spine as it is responsible for lower backache. Due to heavy mechanical stress, slip often occurs at L4 to L5 or L5 to S1. In the past, many approaches have been applied on the vertebrae to detect, segment, and identify various diseases, but, still, researchers are working on better and new techniques to diagnose the diseases more efficiently.

In [15], researchers worked to detect the lumbar spinal stenosis (MRI) images. They worked on axial view of the images and applied SegNet with different training ratios. Mabarki et al. [16] worked on convolutional neural networks (CNN) based on Visual Geometry Group 19 (VGG19) architecture to detect the herniation in the lumbar disc. They tested the system successfully with more than 200 patients. Ala et al. [17] developed a system to find the herniation in disc by taking centroid distance function as a shape feature. They concluded that this feature can be visualized as the best indicator of disc herniation in MRI scan axial images. In [18], authors worked on the mid-sagittal view of the MRI images. They use two segmentation techniques: The first technique was a customized algorithm and the other was semantic segmentation. They obtained good results in classification of spondylolisthesis and lumbar lordosis.

A cascaded fully connected network (FCN) was developed in [19]. They trained the 3D FCN to obtain the lumbar shape and called it a localization net, and then they trained another 3D FCN to segment the cropped lumbar, and called it a segmentation net. The localization net helped the segmentation net to segment the lumbar region correctly. Their results are pretty good, with a dice coefficient of 95%. Liao et al. [20] worked on arbitrary CT images which is a demanding task. As all the images have different shapes and appearances, it is very difficult to segment and localize vertebrae. Therefore, they solved the problem by working on short-range contextual information and long-range contextual information. For short-range contextual information, a 3D FCN is used to extract the features, and for long-range contextual information, they used the bidirectional recurrent neural network, which is applied to encode the contextual information. In conclusion, their method extracts better feature representation than previously used methods with a notable margin on the Medical Image Computing and Computer-Assisted Intervention (MICCAI) Challenge dataset. To predict the centroid coordinates of vertebrae, a deep network was deployed in [21]. They used the public dataset of CT volumetric images and obtained accuracy up to 90%.

Glocker et al. [22] developed a novel approach based on a regression tree. They used two datasets and have a total of 424 CT scans images with different pathologies. Each classification forest is trained to a maximum depth of 24 trees and consists of 20 trees. Their approach works better than regression forest + hidden Markov model (HMM) on pathological spine CT. Pisov et al. [23] worked on a publicly available dataset of the chest to detect the early stage of osteoporosis. Another two-step algorithm is proposed by them, which is used to localize the vertebral column in 3D CT images and the next step is to detect each vertebra and look for fractures in 2D. They trained neural networks for both steps on GPU using an easy six-keypoint-based annotation scheme. Their error is very small, up to 1 mm with very high accuracy up to 0.99. In [24], authors presented their work at Large Scale Vertebrae Segmentation Challenge (VerSe) in 2019, where they used a human–machine hybrid algorithm, with 95% of high vertebrae identification rate and 90% dice coefficient. They used three steps to identify vertebrae: The first step is to detect vertebrae, the second step is to label the vertebrae that is based on btrfly-Net [25], and the third step is to segment vertebrae, which was performed by U-Net. In [26], vertebrae segmentation and labeling was carried out by using a FCN. They segmented the vertebrae by combining the network with a memory component that keeps information about already-segmented vertebrae. After segmentation, it then searches for another vertebrae that is located next to the segmented one and predicts whether it is visible enough to process for further analysis. The methodology attained very high accuracy of 93%, with only one mislabeled vertebrae case. Lecron et al. [27] tried to develop an automatic approach to detect the vertebra. The purpose of developing such model is to detect vertebra without human involvement. They obtained the points of interest in radiography by an edge polygonal approximation, and a scale-invariant feature transform (SIFT) descriptor was used to train a support vector machine (SVM) model. They conclude that their results are very promising, with a corner and vertebrae detection accuracy rate up to 90% and 86%. James et al. [28] proposed a system to detect and localize vertebrae. It detects vertebrae using 3D samples and identifies the specific vertebrae using 2D slices. Their results show very accurate identification and localization of vertebrae.

Friska et al. [29] developed an automated system to measure the foraminal widths and anteroposterior diameter to determine the disease called lumbar spinal stenosis. They used SegNet to obtain six regions of interests in composite axial MRI Images. The results reported 97% agreement with the specialists’ opinion to identify the severity in the intervertebral disc herniation. Boundary detection method using dynamic programming was developed in [30]. They calculated the Euclidean distance between their method of detecting the boundary and manual labeling of lumbar spine and achieved the mean Euclidean distance of 3 mm. Ghosh et al. [31] proposed a system that uses two methods to detect and localize the intervertebral disc (IVD). The system detects IVD by using different machine learning algorithms and segments all the tissues in lumbar sagittal MRI by using different features and training them on robust classifiers. The process achieved promising results with both methods. Gang et al. [32] proposed a novel approach of adding three CNN layers in You Look Only Once (YOLO)-tiny. Their system was used to detect spinal fractures with accuracy of 85.63%. Zuzanna et al. [33] used YOLOv3 to detect different regions in the pelvic area. Modified YOLOv3 is developed in [34]. The researchers used the approach to locate the IVD and detect disc herniation.

Bagus Adhi Kusuma [35], in his research article, addressed the detection of scoliosis using X-ray images. The author preprocessed by converting X-ray images to grayscale and marked seed locations that divide images into 12 sub-images. Later, median filtering and canny were applied to obtain the boundary or vertebrae. After center point calculations, polynomial curve fitting, and Cobb angle estimation, with the help of gradient equation, was achieved. K-mean clustering played a significant role to determine the scoliosis curve. The procedure average deviation is less than 6 degrees. Yaling Pan et al. in [36] used two separate mask regions with convolutional neural network (R-CNN) models to segment and detect the spinal curve and all vertebral bones on 248 X-rays. The Cobb angle is measured from the output of these models. Measuring the angle between any interior and superior perpendicular of the cranial and caudal vertebrae, a set containing all possible angles is obtained, and a maximum angle is considered as the Cobb angle. To assess the reliability and accuracy, two experienced radiologists separately measured the Cobb angle. Manually output results of these models were compared, achieving intraclass and interclass correlation coefficients of 0.941 and 0.887, respectively.

In [37], Safari et al. developed a semi-manual approach for the estimation of Cobb angle. Contract stretching is used to extract the ROI in an input X-ray image. The curvature of the spine is determined with the help of manual landmarking of at least one point for each vertebra, and a fifth-order polynomial curve fitting is applied. After determining the morphologic curve, the final phase is to estimate the Cobb angle by using a tangent equation. The equation is calculated at the inflection points, and the angle is between two perpendicular lines to the spinal curve. The paper claims the correlation coefficient between the angle values is 0.81. In [38], a new, high-precision regression technique, adaptive error correction net (AEC-Net), is introduced for evaluation of Cobb angle from X-ray images of spine. The proposed technique has two modules: The first one is regressing landmark net for boundary features extraction that indirectly aids in Cobb angle calculation. The second one is angle net for direct approach for Cobb calculation using curve features. The final stage is error correction net that basically estimates both modules’ output using extrapolation to identify the difference in Cobb angles from both networks. To evaluate the results, 581 spinal anterior–posterior X-ray images were utilized, attaining a mean absolute error of 4.90 in Cobb angle.

Kang Cheol Kim et al., in [39], presented an approach to identify scoliosis from X-ray images; they explained the drawbacks of manual measurements which are laborious and time-consuming. The method consists of three major parts: in the first part, a confidence map is utilized for localization. In the second part, a vertebral-tilt field is used for the estimation of slope of each vertebra, and in the third part, the Cobb angle is measured using vertebral centroids in combination with the calculated vertebral-tilt field. The performance is evaluated, accomplishing circular mean absolute error (CMAE) of 3:51 degree and symmetric mean absolute percentage error (SMAPE) of 7:84% for the Cobb angle. The main purpose of these works are to aid the clinicians in handling the time-consuming task of manual image labeling.

The researchers have utilized different image processing and machine learning techniques for analysis of spine to identify different lumber deformities. Recently, utilization of deep learning has also been carried out for this purpose. The automated analysis of lumbar deformities relies on accurate localization of vertebrae, and even a small variation in the centers can lead to false grading of deformities. In the current state-of-the-art approaches, almost no research has been carried out on the localization of vertebrae. Most of them have taken this problem as segmentation, which generally faces challenges in the presence of noise and illumination changes. With recent advancements in deep learning, we have more robust object localization techniques which are invariant to these changes, so these techniques can be utilized for localization of vertebrae and further analysis of spinal deformities. Keeping all these gaps and challenges in mind, the contributions made in this research work are as follows:

1. This paper presents the object detection framework for lumbar deformities and provides the research community an annotated dataset in the sagittal plane with labels in YOLO format.
2. One of the major contributions of this research work is to utilize the object detection/localization module as vertebrae localization in comparison to current state-of-the-art methods which are based on semantic segmentation.
3. Edge-based segmentation is used to obtain the localized vertebrae to diagnose the disease.
4. Furthermore, we provide automated methods to calculate the angles to diagnose lumbar deformity, such as lumbar lordosis, and its further grading, which will be used as a decision support system for young radiologists and helps them to grade the severity of lumbar deformities.

## 3. Materials and Methods

### 3.1. Materials

**Figure 2:**
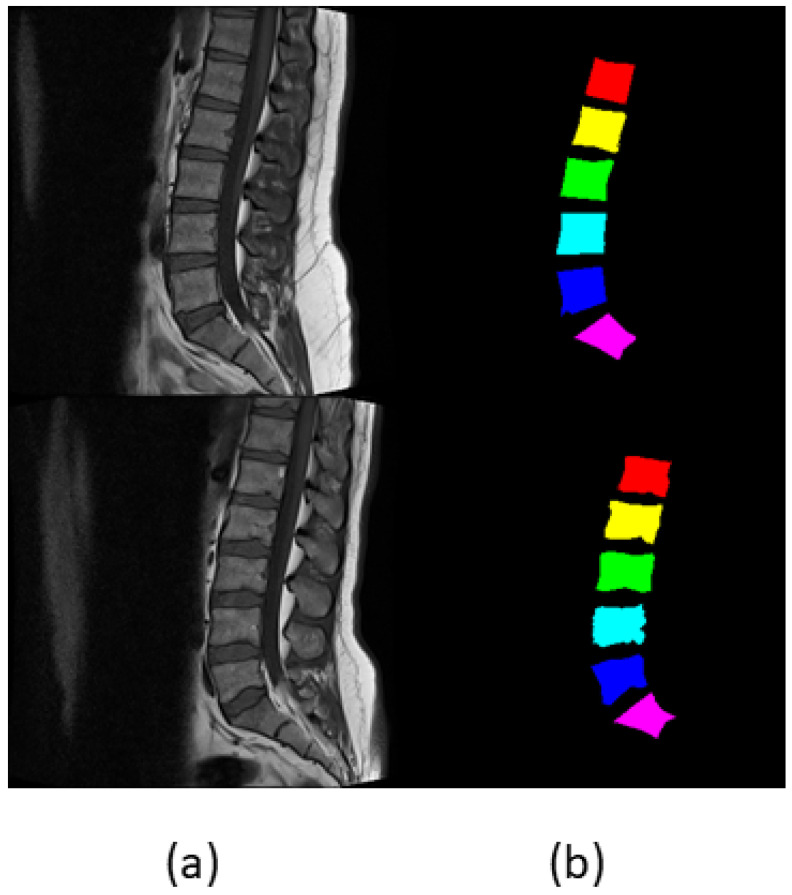
(**a**,**b**) represent the mid-sagittal view of the lumbar spine with its respective pseudo-colored labels.

A number of datasets have been developed by hospitals and challenges to diagnose the spinal deformities. MRI Dataset [40] was collected from patients who reported symptomatic back pain between September 2015 and July 2016 at the Irbid Speciality Hospital in Jordan. Ref. [15] collected the MRI scans of 575 subjects but removed 60 scans due to noise and distortion. They were sure to keep the MRI scans pf patients with age of at least 17, so the lumbar spines have the same physiology. Ref. [40] has annotations of axial views and it has both T1 weighted and T2 weighted scans. This research was performed on Lumbar Spine Composite Dataset [41], which is posted on Mendeley Data, and it contains sagittal views of MRI images of 514 subjects. Lumbar Spine Composite Dataset is originally taken from MRI Dataset, while [18] annotated Lumbar Spine Composite Dataset into mid-sagittal views for effectiveness of results. Ref. [40] has data of 515 subjects, while [41] discarded one subject due to noisy picture and has data of 514 subjects, with ground truth labels, marked pixelwise, and pseudo-colored labels available for the segmentation. According to [42], bone shows the same contrast for both T1 and T2 weighted scans, therefore [18] applied an empirical evaluation and selected either type for spinal measurement. It also includes spinal measurements, ground truth labels where L1, L2, L3, L4, L5, and S are marked pixelwise, and pseudo-colored ground truth images. Spinal measurements help surgeons in suggesting and selecting the appropriate surgical procedure. The resolution of the images and labels are 320 × 320. Figure 2 shows images from composite dataset.

**Figure 3:**
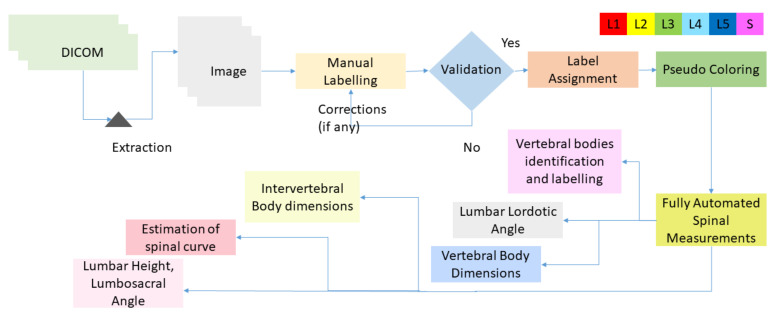
Flow diagram representing the extraction of images, manual labeling and validation, pseudo-coloring, and performing automated spinal measurements.

Figure 3 shows the following steps on MRI Dataset to acquire composite data:

1. Dicom viewer was used to read the image and mid-sagittal view was exported from MRI Dataset.
2. Images were manually labeled from lumbar spine to first sacrum vertebra after obtaining the sagittal view.
3. Labeled regions were consulted with radiologists and validated by expert surgeons.
4. Labels were assigned and pseudo-coloring was applied where each vertebra is represented by different color.
5. Fully automated spinal measurements were performed that contain lumbar lordotic angle (LLA), lumbosacral angle (LSA), dimensions of lumbar spine and sacrum and their identification and labeling, height of lumbar spine, dimensions of discs, and spinal curve estimation.

### 3.2. Methods

**Figure 4:**
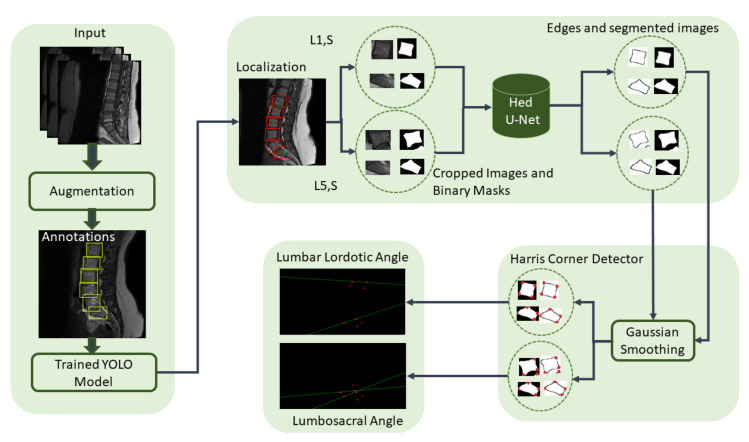
Proposed Framework: Dataset is augmented, annotated, and passed through the YOLOv5 to obtain localized vertebrae. L1 and S for LLA and L5 and S for LSA are extracted from the localized vertebrae, which are then passed through the HED U-Net to obtain the edge-based segmentation. Images are smoothed using Gaussian smoothing filter, and Harris corner detector is applied to obtain the corners of desired vertebrae. LLA and LSA can be found from the corners.

Spine deformity can be due to many reasons, but severity in the disease can led to the lifetime paralysis, and localization and segmentation techniques can be used to analyze the spine deformities. In this research, an automated lumbar spine analysis was performed to diagnose the lumbar spine deformities. Our proposed approach is divided into two different techniques: in the first step, vertebrae are localized by using the object detector, while in the second step, the localized vertebrae are passed through the edge-based segmentation model to diagnose the severity of the lumbar spine disease. Figure 4 shows the flow of our proposed methodology where the dataset is localized using YOLOv5 and then passed through holistically-nested edge detection (HED) U-Net [43]. L1 and S for LLA and L5 and S for LSA are extracted from the localized image. Images are smoothed using Gaussian smoothing filter with a sigma value 1, and Harris corner detector [44] is applied to obtain the corners of desired vertebrae. LLA and LSA can be found from the corners.

### 3.3. Localization

Localization identifies the location of the objects in an image and draws a bounding box around the objects [45]. The annotated dataset is passed through YOLOv5 to obtain the localize vertebrae. Bounding boxes across each vertebra are used to crop the images.

#### 3.3.1. Preprocessing

Preprocessing is the main and first step before training the model. Images are auto-oriented and resized to 416 × 416. Other preprocessing steps involved are data augmentation, data labeling, and annotations.

#### 3.3.2. Data Augmentation

Augmentation of the dataset is necessary to reduce overfitting and increase variability in the dataset [46], so the steps involved in augmentation are noise addition, image flipping, 90° rotation, image cropping, and image shearing. The abovementioned steps are discussed below:

Noise: The salt and pepper noise is added to 5% of image pixels.

Flip: Images are flipped to horizontal.

90° Rotate: Images are 90° rotated to clockwise, counterclockwise, and upside down.

Crop: Images are cropped to 0° minimum zoom and 20° maximum zoom.

Rotate: Images are rotated between −23° and +23°.

Shear: Images are sheared to ±15 horizontally and vertically.

Number of images is increased from 514 to 1028 after augmentation. Each image after augmentation has different values of each step.

#### 3.3.3. Data Labeling and Annotations

**Figure 5:**
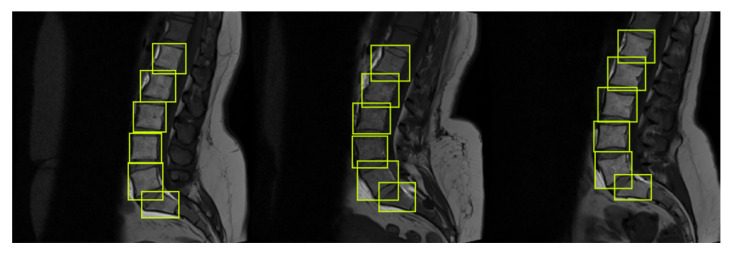
Images are labeled in mid-sagittal view. Each image has the label file of the same name which is generated after labeling the image, and it contains the class, coordinates, and height and width of the objects that are lumbar vertebrae and sacrum bone in this case.

To label the dataset, we used LabelMe and Roboflow Annotate, which is used to label the whole dataset or correct any already-present annotations [47]. Each image contains six annotations of a single class in YOLO format. YOLO format is txt file with the same name as the image that consists of class, *x* and *y* coordinates of object, and width and height of object. Class name is defined as V for vertebrae, and six labels contain five lumbar vertebrae and sacrum in sagittal view, as shown in Figure 5.

#### 3.3.4. Training

The annotated dataset is trained on YOLOv5 with 32 batch size and 90 epochs. We started training the data from epoch 5 and batch size 2 until we obtained the best results. Data is divided into 85% training, 10% validation, and 5% testing. YOLOv5 detected the lumbar vertebrae and sacrum with very good confidence score. YOLO stands for You Look Only Once and was developed in 2015 as an object-detecting system using single neural network that contains multiple convolution networks. The YOLO algorithm became very popular due to its high speed and accuracy. Object detection has been reframed as a single regression problem by YOLO, and this model predicts bounding boxes and class probabilities from image pixels. The YOLO algorithm finds the bounding boxes of objects and probabilities of classes in boxes. Due to its good results in determining and detecting the object coordinates, it stands out more than other object detection algorithms at the time of its release [48].

Average precision is used to evaluate the accuracy of YOLOv5; it calculates the average precision values for over 0 to 1 recall value. *Precision* is defined as the ratio of true positive cases and total number of true predictions.

(1) $$P=\frac{True\;Positive\;Cases}{Total\;Positive\;Predictions}$$

*Recall* is defined as the ratio of true positive cases and the total number of cases.

(2) $$R=\frac{True\;Positive\;Cases}{Total\;Cases}$$

**Figure 6:**
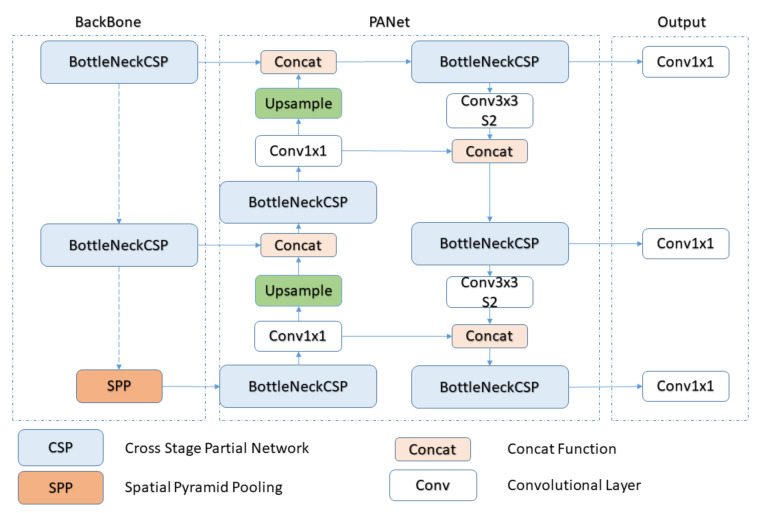
YOLOv5 Network Architecture: It consists of three stages: In the first stage, data is fed into the backbone which extracts the features, then it passes through the neck in the second stage, where the features are fused, and the third stage, named head, outputs the detection results [51].

YOLOv5 has four models: YOLOv5s, YOLOv5m, YOLOv5l, and YOLOv5x. We used the smallest and the fastest model, YOLOv5s. It has a size of 14 Mb and has 2.2 ms inference time. YOLOv5 network architecture is shown in Figure 6. It consists of three stages:

1. CSPNet:Backbone YOLOv5 includes cross-stage partial network (CSPNet) [49] into darknet and makes CSPDarknet its backbone. It decreases the model’s floating-point operations per second (FLOPS) and parameters by solving the problem of repeated gradient information in large scale and integrates the gradient changes into a feature map. This not only reduces the size of the model but also ensures the accuracy and speed of inference.
2. PANet [50]:Neck It increases the flow of information through pipelining. Low-level features can be propagated efficiently by adding a feature pyramid network (FPN), which is a new feature with properties such as bottom-up path augmentation. Feature grid and all other features are linked together by adaptive pooling which makes the useful information in each feature level. The network decides which features are useful from all the layers. It increases the accuracy of object location by using the correct localization signals in lower layers [51].
3. YOLO Layer:Head This is the last layer of YOLOv5, and it detects the results in the form of confidence score, size, and accuracy. It contains three different types of feature maps, i.e., 18 × 18, 36 × 36, and 72 × 72, to detect small, medium, and big objects [51].

### 3.4. Edge-Based Segmentation and Identification

**Figure 7:**
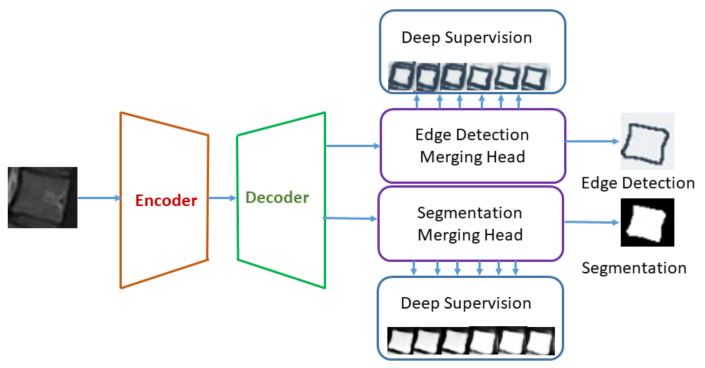
High-level structure of HED U-Net. Encoder and decoder compute the feature maps pyramids and specific task-merging heads unite this information using hierarchy attention maps [43].

Binary masks were created to pass the localized vertebrae through HED U-Net and obtain the segmented images and their detected edges. Extracted images are smoothed and corners are found, and lastly, lines are drawn to find the angles. We cropped L1, L5, and S through the bounding boxes, and cropped images with their binary masks are passed through the HED U-Net. The best results are obtained with the batch size 8, epoch size 10, learning rate 0.001, and optimizer as Adam. Heidler in [43] developed HED U-Net, which is a combination of segmentation and edge-detection framework. They unified the U-Net [52] for semantic segmentation and HED [53] for edge detection in a natural way. In our case of segmentation, there are two class labels, i.e., vertebra and background, and in the case of edge detection, there are two classes, which are “no edge” and “edge”. Figure 7 shows the high-level representation of the HED U-Net; the image is passed through the encoder where downsampling of the image is performed to accumulate the contextual information at low resolution. Then, the decoder upsamples the image by distributing this information to individual pixels. Element-wise addition is used to merge the data flows in the decoder part. In this model, the researchers used six resolution levels of feature pyramid, where the full image resolution has the finest feature map and a poor feature map at resolution 1/32 [43]. The model is deep supervised, where it is trained to predict the ground truth at every level of the feature pyramid. Deep supervision is used to increase the generalization capacity and learning efficacy of a model. Network captures better results after deep supervision and encodes meaningful features present in the deep layers by making use of the available receptive field. After computing the feature pyramid through encoder–decoder methods, two task-specified merging heads are employed which use hierarchy attention mechanism to combine this information. In this mechanism, most useful features of each pixel are focused, rather than fused, features of fix weights [43].

#### 3.4.1. Image Smoothing and Corner Calculation

**Figure 8:**
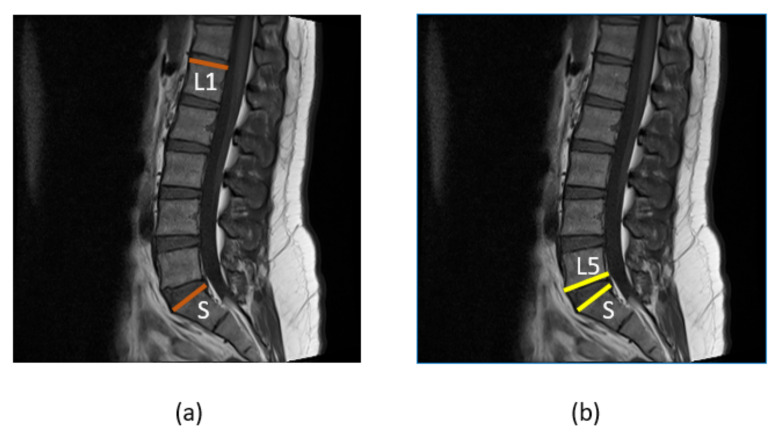
(**a**) Brown lines are drawn on the superior endplates of L1 and S to represent the lumbar lordotic angle (**b**) while yellow lines are drawn on the inferior and superior endplates of L5 and S to represent the lumbosacral angle.

After the images are passed through HED U-Net, we obtain the segmented image and the detected edges. We cropped the images from the bounding boxes we received from YOLOv5. We must compute the LLA and LSA. LLA is the angle between the L1 superior endplate and S superior endplate, while LSA is the angle between the L5 inferior endplate and S superior endplate, as shown in Figure 8, so we used the bounding boxes of L1 and S for LLA and L5 and S for LSA to crop the desire vertebra. To obtain the smooth image, the vertebra images are then passed through the Gaussian smoothing filter with the sigma value equal to 1, which helps us in finding the corners through the Harris corner detector [44] accurately. The Harris corner detector is applied on the smoothed images to obtain the corners of L1 and S for LLA and corners of L5 and S for LSA.

#### 3.4.2. Angles Computation

**Figure 9:**
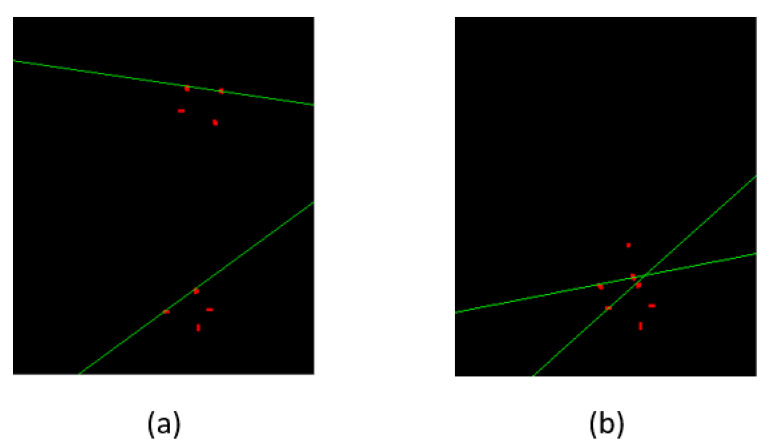
(**a**) Lines are passed through the corners of L1 and S to calculate the LLA (**b**), while lines are passed through the computed corners of L5 and S to calculate the LSA.

In the case of LLA, a line is drawn from the superior corners of L1 and another line is drawn from the superior corners of *S*, while for LSA, one line is drawn from the inferior corners of L5 while another is drawn from the superior corners of *S*, as shown in Figure 9. The slope of the lines can be found from the following formula:

(3) $$m_{L1,S}=\frac{p2_{y}-p1_{y}}{p2_{x}-p1_{x}}$$

where *m* represents the slope of lines, $p1_{y}$ and $p2_{y}$ are *y*-axis points, while $p1_{x}$ and $p2_{x}$ are the *x*-axis of two points. The angle between the lines can be found from the below expression:

(4) $$Angle=tan^{-1}\left|\frac{m_{L1}-m_{S}}{1+m_{L1}m_{S}}\right|$$

where $m_{L1}$ is the slope of superior endplate of L1 line, while $m_{S}$ is the slope of superior endplate of *S* line. The same equations can be used to find the slope and angle for LSA.

### 3.5. Evaluation Metrics

The proposed approaches were trained on RTX 2070 GPU with 16 GB RAM and implemented in Python using Pycharm and Anaconda. We used different Python libraries, some of them are torch, torchvision, PyYAML, and scipy. To measure the performance of our methodology for localization, we used Euclidean distance (EU) and intersection over union (IOU) metrics. Distance between the centroids is calculated by EU. The equation for the EU is given below:

(5) $$\mathrm{EU}=\sqrt{{\left(x_{2}^{.}-x_{1}^{.}\right)}^{2}+{\left(y_{2}^{.}-y_{1}^{.}\right)}^{2}}$$

where $x_{1}^{.}$ and $y_{1}^{.}$ are the centroid coordinates of ground truth boxes, while $x_{2}^{.}$, $y_{2}^{.}$ are the *x*- and *y*-axis of predicted boxes. Each image contains seven EU, one for each class. Smaller value of EU indicates less error of distance between the centroids of predicted bounding box with ground truth box. All the measurements taken on the image are in mm. The overlap of ground truth box and predicted box is measured through IOU. To find the IOU, the following formula is used:

(6) $$\mathrm{IOU}=\frac{AO(Area\;of\;Overlap)}{AU(Area\;of\;Union)}$$

IOU is the ratio between the area of two boxes overlap and the total area of two boxes, as shown in Equation (3). The larger the value of IOU, the more overlap of the two boxes there is. If the value of IOU is 0.95, it means that the two boxes are overlapped by 95 percent. Mean error computes the error between the angles, and it can be found as follows:

(7) $$\mathrm{ME}=\frac{1}{k}\sum_{l=0}^{k-1}\left|a_{pred_{l}}-a_{orig_{l}}\right|$$

ME represents the mean absolute error of angles; its value is in degrees, and it calculates the error in the estimated and predicted angles. Smaller value of ME shows less error between the predicted and ground truth values. K is the total number of images and *a_pred_* is the angle predicted, while *a_orig_* is the ground truth value of an angle.

## 4. Results and Discussion

Various deep learning techniques were applied on the same dataset, but we used object detector for the localization of lumbar spine and sacrum and the labelMe python package for data annotations and saved it in YOLO format. Vertebrae were localized with a very good confidence score. An empirical threshold of 0.65 was applied on confidence score to eliminate the boxes with lower scores. The non-maximum suppression (NMS) intersection over union (IOU) threshold is set to 0.3 in testing data.

**Figure 10:**
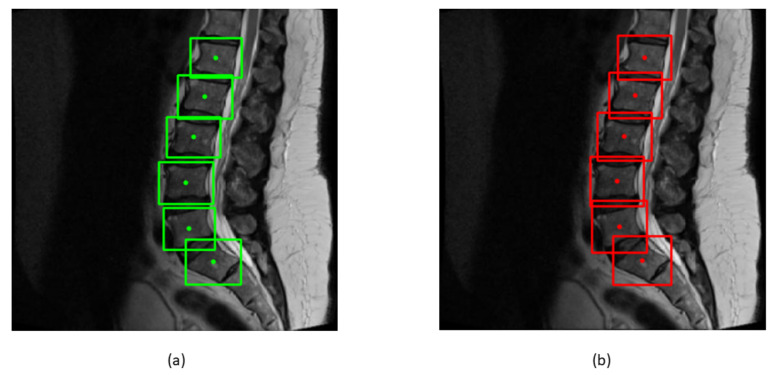
(**a**) Green bounding boxes represent the ground truth boxes with their centroids; (**b**) Red bounding boxes represent the predicted bounding boxes with their centroids.

Bounding boxes are drawn across each vertebra to find the centroids of boxes. The ground truth and predicted centroids are measured by the following formula:

(8) $$Centre_{x,y}=\frac{x_{min},y_{min}+x_{max},y_{max}}{2}$$

where *x_min_*, *x_max_*, *y_min_*, and *y_max_* represent the *x* and *y* coordinates of bounding boxes. The ground truth and predicted centroids are shown in green and red colors in Figure 10.

After training and testing the model, the ground truth and predicted bounding boxes were compared by the EU of center points. The mean of the EU between the centroids of bounding boxes and IOU of two boxes can be calculated from the following formula:

(9) $$M_{\mathrm{EU}}=\frac{\sum_{l=0}^{F-1}{\mathrm{EU}}_{l}}{F}$$

Table 1 shows the mean and the standard deviation of six vertebrae. The distance between the centroids is in millimeters; the sacrum has the highest EU mean due to its tilted structure, while L1 has the least EU mean of 1.6 mm. Low value of mean indicates less distance between the centroids. Table 2 shows the mean and standard deviation of IOU of each vertebra. Each vertebra has high value of mean and low value of standard deviation. High value of mean shows higher overlap between the bounding boxes.

**Figure 11:**
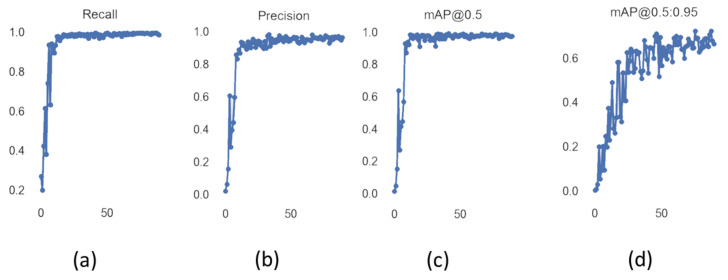
(**a**,**b**) represent the recall and precision values of YOLOv5s during training with increasing epochs; (**c**) mAP@0.5 represents mAP values with the IOU threshold value as 0.5; (**d**) mAP@0.5:0.95 represents mAP values with different IOU threshold values that range from 0.5 to 0.95 with step size of 0.05.

**Figure 12:**
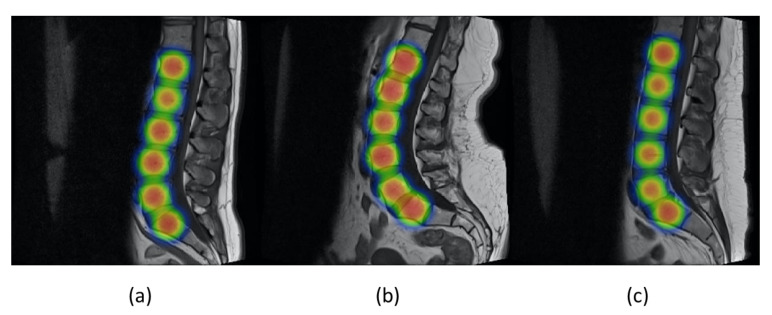
(**a**–**c**) represent the YOLOv5 visualization in heat maps obtained from its weights after training on the three different images.

Figure 11 shows the values of precision and recall of object detector YOLOv5 with increasing values of epoch from 0 to 90. Mean average precision (mAP) is the mean of average precision, and we obtained mAP of 0.975 by using YOLOv5s. YOLOv5 can be visualized in heat maps from its trained weights before applying non-maximum suppression, as shown in Figure 12.

**Figure 13:**
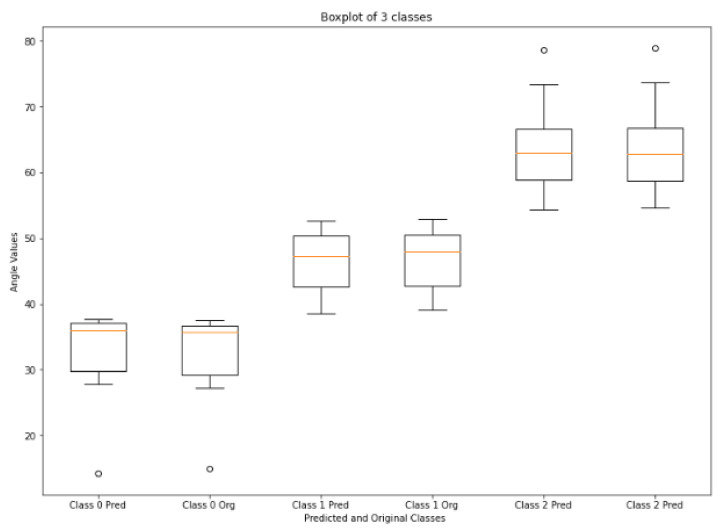
Boxplot of hypo, normal, and hyper lumbar lordosis. All the predicted and original angle values of each class lie in the same range.

Figure 13 shows the boxplot values of calculated and ground truth angles of three classes calculated from the corners of vertebrae that are passed through HED U-Net. Normal LLA ranges from 39° to 53° [54], so angles less than 39° are in the range of hypolordosis, named as class 0. Normal lordosis is class 1, and class 2 is hyperlordosis, having angle values above 53°. The boxplot shows very small error between the calculated values and ground truth values of angles. According to [55,56,57], the measurement error up to ±3°–±5° is clinically accepted. The mean error and standard deviation of mean error of LLA and LSA are shown in Table 3. LLA and LSA calculated from our method have very small mean error values, i.e., 0.29° and 0.38°. A confusion matrix for this technique is shown in Table 4; low values of mean error indicate there is no failure cases in any class. It has classified all the 51 subjects of lordosis correctly.

### 4.1. Comparison of Models

We compared YOLOv5 with other object detection models in Table 5. Region-based fully convolutional network (R-FCN) [58] has residual networks (ResNet) as a backbone to detect the objects. We achieved 0.894 mAP value to detect the lumbar vertebrae and a sacrum by using R-FCN. SSD513 [59] is the single shot detector with 513 × 513 inputs that detects the vertebrae with the mAP of 0.925. Lin et al. [60] used feature pyramid networks (FPN) architecture in faster RCNN to detect the object more accurately. We obtained mAP value of 0.942 by using the FPN FRCN, which is the second highest value in Table 5. YOLOv3 is the third variant of the YOLO family, which is believed to be three times faster than SSD [61]. It achieved 0.917 mAP in detecting the vertebrae. YOLOv5 has surpassed other current state of the art methods and its mAP is 0.033 greater than FPN FRCN.

### 4.2. Comparison of Approaches for Lumbar Lordosis Assessment

**Figure 14:**
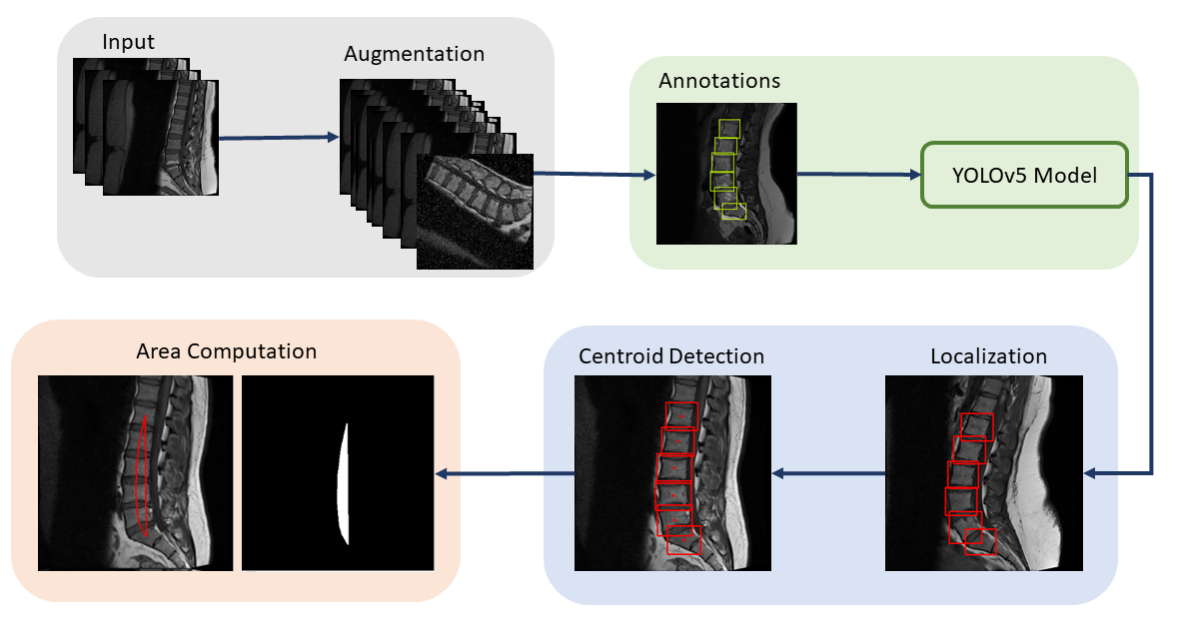
Region-area-based lumbar lordosis identification block diagram. The preprocessed dataset is passed through the localization network with its labels. Vertebrae are localized with very good confidence score and its centroids are calculated and joined together to form a region whose area can be calculated to find the severity of lumbar lordosis.

We used two ways to diagnose the lumbar lordosis. This first method is to assess the lumbar lordosis by finding the angle from the corners of the vertebra, while the other method is the lumbar lordosis assessment through region area, where the area is correlated with the angles values to classify the disease. A block diagram of the region-area-based method to diagnose the lumbar lordosis is shown in Figure 14. The presented technique consists of preprocessing steps, training of YOLOv5 model, vertebrae localization, centroids calculations, and area computation through centroids. The centroids of localized vertebrae can be connected together to find the area of enclosed region, which will be used to diagnose the severity of the disease.

Ref. [18] proposed the method to diagnose lordosis on the basis of area enclosed in the region. They combined [62,63] techniques to obtain the area under the curve from the centroids. We computed the area from the centroids of the bounding boxes of YOLOv5. The centroid of L1 is connected to L2, L2 to L3, and so on, and lastly, the centroid of S is connected to L1, to form an enclosed region. The normal range for lordosis is 39° to 53° [54]. Angles below 39 are termed as hypolordosis, while the angles above 53° are termed as hyperlordosis. The area of the enclosed region is correlated with the angles to diagnose the disease. The area can be found by summing all the non-zero pixels in the images. The equation is given by

(10) $$Area_{region}=\sum_{i=0}^{total_{n}}zz_{i}\times \Delta zz$$

**Figure 15:**
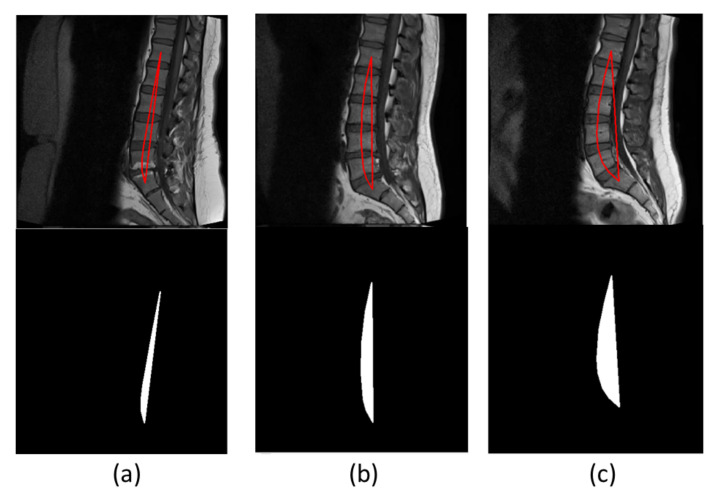
Each column represents the region plot of an image and its respective area computation image. (**a**–**c**) represent the hypolordosis, normal lordosis, and hyperlordosis cases, respectively. Each image has different curve and area which differentiates the disease.

*Area_region_* represents the area of the region, *zz_i_* is the non-zero pixels in the images, and Δ*zz* is the interval between pixels and is equivalent to one pixel. This method was proposed by [18] to diagnose the lordosis from the centroids instead of corner points. Figure 15 shows three images, each from a different lordosis type. Hypolordosis includes straight back and flat back cases, while hyperlordosis includes sway back of the vertebrae. The region area of normal lordosis is smaller than the hyperlordosis and greater than the hypolordosis curve [18]. We obtained 74.5 percent accuracy by using this technique. Table 6 shows the confusion matrix for this technique; the total number of test cases is 51, in which 6 subjects have hypolordosis, 19 subjects have normal lordosis, and 26 subjects have hyperlordosis. Hypolordosis is classified correctly, while normal and hyper have some of the misclassified cases.

The summary of all the results is shown in Table 7. The results show that lumbar assessment through corners classifies the class more accurately as compared to lumbar lordosis assessment through region area. Our proposed approach outperforms the region-area-based approach by 25.5%. The first method uses the localization and edge-based segmentation methods to diagnose the disease, while the region-area-based method uses only the localization part and then calculates the centroids of the vertebrae to calculate the area of the enclosed region. Accuracies for both techniques are shown in Table 7.

### 4.3. Comparison with Other Researchers

We also compared our proposed model with other researchers. Our proposed methodology uses YOLOv5 for the localization and HED U-Net for the edge based segmentation, Masood et al. used Resnet-UNet for the segmentation of lumbar vertebrae, Suri et al. [64] used three neural networks, each network for each modality of image to compute the LLA, while Cho et al. [65] used semantic segmentation by using the model UNet We obtained very small mean error of LLA and LSA as compared to [41,64,65]. The first two techniques are applied on Composite Lumbar Spine MRI Dataset [41]. As we can see from the results, our object detection and edge-based segmentation model outperforms other state-of-the-art models that use semantic segmentation. This system will help the clinicians and support their decisions in diagnosing the disease. Table 8 shows the comparison of our results with other researchers. Our proposed technique achieves ME of 0.29° and 0.38° for LLA and LSA, which is 2.32° and 1.63° less than the ME of LLA and LSA calculated by the proposed approach of Masood et al.

Clinicians often use time-consuming different manual or semi-manual methods to calculate the Cobb angles and diagnose the vertebral diseases. Our proposed method is an automated method to compute the Cobb angles with very small mean error value. According to [66], clinicians take around 18.96 s to calculate the Cobb angles, while our method gives the Cobb angles results within 5 s on 10th gen Intel core i7 and Nvidia Geforce RTX 3060. We used the object detector to localize the vertebrae, which has not been used before, and then edge-based segmentation to diagnose the disease severity. As no object detection framework for diagnosing the spinal disorder is present, the comparison is difficult. We also compared YOLOv5 with other object detectors, i.e., R-FCN, SSD513, FPN FRCN, and YOLOv3. We achieved the highest value of mAP by using the YOLOv5 model. The region-area-based method tends to fail because it is dependent on centroids of multiple vertebrae, and small error in the computation of the centroids can affect the whole area, which can led to misclassification. Cobb angle is dependent on the end plate structures of vertebrae, and its computation often tends to fail if there is an abnormal-shaped vertebra in test phase, as the model has been trained on the dataset having mostly healthy vertebrae. Our primary focus is to develop a fully automated system to diagnose the disease, and very little attention is given to the abnormally deformed vertebrae.

## 5. Conclusions

In this paper, we performed the YOLO annotations of the Lumbar Spine Composite Dataset [41], which consists of the mid-sagittal views of MRI scans. We proposed a method to localize the lumbar spine and a sacrum using YOLOv5 and performed the calculations to compute the centroids of bounding boxes. The centroids are compared with ground truth centroids values which have very low mean error and high IOU in the case of each lumbar and sacrum vertebra. Region area computed from the centroids decides the lumbar lordosis severity, i.e., sway back, normal, or flat back, which will help the young doctors as a decision support system to diagnose the disease. Localized vertebrae are passed through HED U-Net to obtain the vertebrae and their edges. The angles, LLA, and LSA are found by computing the corners of vertebra using Harris corner detector with a very small mean error and standard deviation. It is concluded from the experiments that images should be smoothed before finding the corners of vertebrae; unsmoothed images make corner detection a laborious task. For this purpose, a Gaussian smoothing filter with a sigma value 1 was used to obtain the smoothed corners. LLA has 0.28° mean error which means it detects the lumbar lordosis, hypo, normal, and hyper, very efficiently. In the future, this work can be extended to diagnose cervical, thoracic spine, and pelvic region deformities. Other directions may be used to investigate and develop a fully automated machine learning toolkit for spinal deformities to prevent invasive surgery methods.

## Figures and Tables

**Table 1:** This table represents the mean and standard deviation of EU. L1 has the lowest value of mean and L6 has the highest value of mean among all the vertebrae.

Vertebrae	EU Mean Values	EU Standard Deviation Values
L1	1.665	1.1468
L2	2.233	1.1826
L3	2.4287	1.3533
L4	2.142	1.0888
L5	2.520	1.7808
S1	2.983	1.0894

**Table 2:** This table represents the mean and standard deviation of IOU. L1 has the highest value of IOU mean with lowest value of std.

Vertebrae	IOU Mean Values	IOU Standard Deviation Values
L1	0.878	0.0465
L2	0.873	0.056
L3	0.859	0.055
L4	0.8616	0.053
L5	0.848	0.067
S1	0.823	0.0619

**Table 3:** This table represents the mean error and std of LLA and LSA. LLA has lower mean error and std than LSA mean error and its std.

Angles	Mean Values	Standard Deviation Values
LLA	0.878	0.0465
LSA	0.873	0.056

**Table 4:** Confusion matrix of lumbar lordosis assessment through corners of vertebra. There are no failure cases in any of the predicted classes due to less error between ground truth and predicted angles values.

	Hypo	Normal	Hyper
Hypo	6	0	0
Normal	0	19	0
Hyper	0	0	26

**Table 5:** This table represents the comparison of different models. YOLOv5 has the highest mAp value, which means it outperforms other object detectors.

S.No	Models	mAP
1	R-FCN	0.894
2	SSD513	0.925
3	FPN FRCN	0.942
4	YOLOv3	0.917
5	YOLOv5	**0.975**

**Table 6:** Confusion matrix of Lumbar lordosis assessment through region area. Hypolordosis has no failure cases, while normal lordosis has eight, and hyperlordosis has five failure cases. Six cases of normal lordosis lie in hypo, while two cases lie in hyperlordosis. Five failure cases of hyperlordosis lie in normal lordosis.

	Hypo	Normal	Hyper
Hypo	6	0	0
Normal	6	11	2
Hyper	0	5	21

**Table 7:** Comparison table of two methods that were used to diagnose the lumbar lordosis. Lumbar lordosis assessment through corners outperforms the lumbar lordosis assessment through region area. We used the same localization part for both methods; therefore, the value of mAP in both methods is the same.

Methods	Hypo		Normal		Hyper		Mean Error		mAP	Accuracy
	True	False	True	False	True	False	LLA	LSA		
Overall Proposed Methodology	6	0	19	0	26	0	0.29°	0.38°	0.975	100%
Localization & Identification	6	0	11	8	21	5	-	-	0.975	74.5%

**Table 8:** LLA and LSA mean errors for our proposed technique are 0.29° and 0.38°, respectively, while [18] has mean errors of 2.61° and 2.01° for LLA and LSA. The first two techniques are applied on the same dataset. Cho et al. [65] and Suri et al. [64] have relatively larger LLA mean errors.

	Modalities	Techniques	mAP	Mean Error	
				LLA	LSA
Our proposed technique	MRI	YOLOV5-HED-UNET	0.975	0.29°	0.38
Masood et al. [18]	MRI	Resnet-UNET	-	2.61°	2.01°
Suri et al. [64]	MRI	Neural Networks	-	2.90°	-
	CT		-	2.26°	-
	Xray		-	3.60°	-
Cho et al. [65]	Xray	UNET	-	8.05°	-

## Data Availability

Lumbar Spine Composite Dataset used in this study is openly available in Mendeley Data at https://doi.org/10.17632/k3b363f3vz.2, reference number [41].

## Abbreviations

YOLO	You Only Look Once
mAP	Mean average precision
HED	Holistically-nested edge detection
LLA	Lumbar lordotic angle
LSA	Lumbosacral angle
WHO	World Health Organization
MRI	Magnetic resonance imaging
CT	Computerized tomography
ML	Machine learning
AI	Artificial intelligence
FCN	Fully convolutional networks
MICCAI	Medical Image Computing and Computer Assisted Intervention
HMM	Hidden Markov model
VerSe	Large Scale Vertebrae Segmentation Challenge
SVM	Support vector machines
SIFT	Scale-invariant feature transform
CNN	Convolutional neural network
IVD	Intervertebral disc
AEC-Net	Adaptive error correction net
CMAE	Circular mean absolute error
SMAPE	Symmetric mean absolute percentage error
GPU	Graphics processing unit
CSPNet	Cross stage partial network
VGG19	Visual Geometry Group 19
FPN	Feature pyramid network
NMS	Non-maximum suppression
IOU	Intersection over union
ME	Mean error
SSD	Single shot detector
R-FCN	Region-based fully convolutional network

## References

[1] who.int. https://www.who.int/news-room/fact-sheets/detail/spinal-cord-injury.

[2] Armour B.S., Courtney-Long E.A., Fox M.H., Fredine H., Cahill A. (2016). Prevalence and causes of paralysis—United States, 2013. Am. J. Public Health.

[3] webmd.com. https://www.webmd.com/back-pain/ss/slideshow-low-back-pain-overview.

[4] kenhub.com. https://www.kenhub.com/en/library/anatomy/lumbar-vertebrae.

[5] spine-health.com. https://www.spine-health.com/conditions/lower-back-pain.

[6] acatoday.org/. https://www.acatoday.org/Patients/What-is-Chiropractic/Back-Pain-Facts-and-Statistics.

[7] Dashti S., Dashti S.F. (2020). An expert system to diagnose spinal disorders. Open Bioinform. J..

[8] Abdullah-Al-Zubaer I., Huang C., Tang H., Fan W., Cheung K.M.C., To M., Qian Z., Terzopoulos D. (2020). Analysis of scoliosis from spinal X-ray images. arXiv.

[9] Dilsizian S.E., Eliot L.S. (2014). Artificial intelligence in medicine and cardiac imaging: Harnessing big data and advanced computing to provide personalized medical diagnosis and treatment. Curr. Cardiol. Rep..

[10] Patel V.L., Shortliffe E.H., Stefanelli M., Szolovits P., Berthold M.R., Bellazzi R., Abu-Hanna A. (2009). The coming of age of artificial intelligence in medicine. Artif. Intell. Med..

[11] Jiang F., Jiang Y., Zhi H., Dong Y., Li H., Ma S., Wang Y., Dong Q., Shen H., Wang Y. (2017). Artificial intelligence in healthcare: Past, present and future. Stroke Vasc. Neurol..

[12] pngkey.com. https://www.pngkey.com/detail/u2w7r5o0e6t4a9a9_structure-of-spine-human-spine-png/.

[13] josephspine.com. https://josephspine.com/mri-vs-ct-scan-diagnosing-spine-neck-injuries-degenerative-diseases/.

[14] Tang X. (2019). The role of artificial intelligence in medical imaging research. BJR Open.

[15] Al-Kafri A.S., Sudirman S., Hussain A., Al-Jumeily D., Natalia F., Meidia H. (2019). Boundary delineation of MRI images for lumbar spinal stenosis detection through semantic segmentation using deep neural networks. IEEE Access.

[16] Mbarki W., Bouchouicha M., Frizzi S., Tshibasu F., Farhat L.B., Sayadi M. (2020). Lumbar spine discs classification based on deep convolutional neural networks using axial view MRI. Interdiscip. Neurosurg..

[17] Al Kafri A.S., Sudirman S., Hussain A.J., Fergus P., Al-Jumeily D., Al Smadi H., Khalaf M., Al-Jumaily M., Al-Rashdan W., Bashtawi M. (2017). Detecting the Disc Herniation in Segmented Lumbar Spine MR Image Using Centroid Distance Function. Proceedings of the 2017 10th International Conference on Developments in eSystems Engineering (DeSE).

[18] Masood R.F., Taj I.A., Khan M.B., Qureshi M.A., Hassan T. (2022). Deep Learning based Vertebral Body Segmentation with Extraction of Spinal Measurements and Disorder Disease Classification. Biomed. Signal Process. Control..

[19] Janssens R., Guodong Z., Guoyan Z. (2018). Fully automatic segmentation of lumbar vertebrae from CT images using cascaded 3D fully convolutional networks. Proceedings of the 2018 IEEE 15th International Symposium on Biomedical Imaging (ISBI 2018).

[20] Liao H., Addisu M., Jiebo L. (2018). Joint vertebrae identification and localization in spinal CT images by combining short-and long-range contextual information. IEEE Trans. Med. Imaging.

[21] Yang D., Xiong T., Xu D., Huang Q., Liu D., Zhou S.K., Xu Z., Park J., Chen M., Tran T.D. (2017). Automatic vertebra labeling in large-scale 3D CT using deep image-to-image network with message passing and sparsity regularization. International Conference on Information Processing in Medical Imaging.

[22] Glocker B., Zikic D., Konukoglu E., Haynor D.R., Criminisi A. (2013). Vertebrae localization in pathological spine CT via dense classification from sparse annotations. International Conference on Medical Image Computing and Computer-Assisted Intervention.

[23] Pisov M., Kondratenko V., Zakharov A., Petraikin A., Gombolevskiy V., Morozov S., Belyaev M. (2020). Keypoints Localization for Joint Vertebra Detection and Fracture Severity Quantification. International Conference on Medical Image Computing and Computer-Assisted Intervention.

[24] Sekuboyina A., Bayat A., Husseini M.E., Loffler M., Rempfler M., Kukacka J., Tetteh G., Valentinitsch A., Payer C., Urschler M. (2020). VerSe: A Vertebrae Labelling and Segmentation Benchmark. arXiv.

[25] Sekuboyina A., Rempfler M., Kukacka J., Tetteh G., Valentinitsch A., Kirschke J.S., Menze B.H. (2018). Btrfly net: Vertebrae labelling with energy-based adversarial learning of local spine prior. International Conference on Medical Image Computing and Computer-Assisted Intervention.

[26] Lessmann N., Van Ginneken B., De Jong P.A., Isgum I. (2019). Iterative fully convolutional neural networks for automatic vertebra segmentation and identification. Med. Image Anal..

[27] Lecron F., Mohammed B., Saïd M. (2012). Fully automatic vertebra detection in X-ray images based on multi-class SVM. Medical Imaging 2012: Image Processing.

[28] McCouat J., Ben G. (2019). Vertebrae detection and localization in CT with two-stage CNNs and dense annotations. arXiv.

[29] Natalia F., Meidia H., Afriliana N., Young J.C., Yunus R.E., Al-Jumaily M., Al-Kafri A., Sudirman S. (2020). Automated measurement of anteroposterior diameter and foraminal widths in MRI images for lumbar spinal stenosis diagnosis. PLoS ONE.

[30] Koh J., Chaudhary V., Jeon E.K., Dhillon G. (2014). Automatic spinal canal detection in lumbar MR images in the sagittal view using dynamic programming. Comput. Med. Imaging Graph..

[31] Ghosh S., Vipin C. (2014). Supervised methods for detection and segmentation of tissues in clinical lumbar MRI. Comput. Med. Imaging Graph..

[32] Sha G., Junsheng W., Bin Y. (2020). Detection of Spinal Fracture Lesions Based on Improved YOLO-tiny. Proceedings of the 2020 IEEE International Conference on Advances in Electrical Engineering and Computer Applications (AEECA).

[33] Zuzanna K., Jacek S. Bones detection in the pelvic area on the basis of YOLO neural network. Proceedings of the 19th International Conference Computational Problems of Electrical Engineering.

[34] Zhong Z., Jianzhi D. Real-Time Detection based on Modified YOLO for Herniated Intervertebral Discs. Proceedings of the 2019 4th International Conference on Intelligent Information Processing.

[35] Kusuma B.A. Determination of spinal curvature from scoliosis X-ray images using K-means and curve fitting for early detection of scoliosis disease. Proceedings of the 2017 2nd International conferences on Information Technology, Information Systems and Electrical Engineering (ICITISEE).

[36] Pan Y., Chen Q., Chen T., Wang H., Zhu X., Fang Z., Lu Y. (2019). Evaluation of a computer-aided method for measuring the Cobb angle on chest X-rays. Eur. Spine J..

[37] Safari A., Parsaei H., Zamani A., Pourabbas B. (2019). A semi-automatic algorithm for estimating Cobb angle. J. Biomed. Phys. Eng..

[38] Chen b., Xu Q., Wang L., Leung S., Chung J., Li S. (2019). An automated and accurate spine curve analysis system. IEEE Access.

[39] Kim K.C., Yun H.S., Kim S., Seo J.K. (2020). Automation of Spine Curve Assessment in Frontal Radiographs Using Deep Learning of Vertebral-Tilt Vector. IEEE Access.

[40] Sudirman S., Al Kafri A., Natalia F., Meidia H., Afriliana N., Al-Rashdan W., Bashtawi M., Al-Jumaily M. (2019). Lumbar Spine MRI Dataset.

[41] Masood R.F., Hassan T., Akram M.U., Taj I.A., Qureshi M.A., Khan M.B. (2021). Composite Dataset of Lumbar Spine Mid-Sagittal Images with Annotations and Clinically Relevant Spinal Measurements.

[42] MRI Interpretation T1 v T2 Images. https://www.radiologymasterclass.co.uk/tutorials/mri/t1_and_t2_images.

[43] Heidler K., Mou L., Baumhoer C., Dietz A., Zhu X.X. (2021). HED-UNet: Combined Segmentation and Edge Detection for Monitoring the Antarctic Coastline. IEEE Trans. Geosci. Remote Sens..

[44] Harris C., Stephens M. (1988). A combined corner and edge detector. Alvey Vis. Conf..

[45] machinelearningmastery.com. https://machinelearningmastery.com/object-recognition-with-deep-learning/.

[46] medium.com. https://medium.com/analytics-vidhya/data-augmentation-is-it-really-necessary-b3cb12ab3c3f.

[47] blog.roboflow.com. https://blog.roboflow.com/labeling/.

[48] Thuan D. (2021). Evolution of YOLO Algorithm and YOLOv5: The State-of-the-Art Object Detection Algorithm. Bachelor’s Thesis.

[49] Wang C.-Y., Liao H.Y.M., Wu Y.H., Chen P.Y., Hsieh J.W., Yeh I.H. CSPNet: A new backbone that can enhance learning capability of CNN. Proceedings of the IEEE/CVF Conference on Computer Vision and Pattern Recognition Workshops.

[50] Wang K., Liew J.H., Zou Y., Zhou D., Feng J. Panet: Few-shot image semantic segmentation with prototype alignment. Proceedings of the IEEE/CVF International Conference on Computer Vision.

[51] Xu R., Lin H., Lu K., Cao L., Liu Y. (2021). A Forest Fire Detection System Based on Ensemble Learning. Forests.

[52] Ronneberger O., Fischer P., Brox T. (2015). U-net: Convolutional networks for biomedical image segmentation. International Conference on Medical Image Computing and Computer-Assisted Intervention.

[53] Xie S., Tu Z. Holistically-nested edge detection. Proceedings of the IEEE International Conference on Computer Vision.

[54] David W.P., Kilkelly F.X., McHale K.A., Asplund L.M., Mulligan M., Chang A.S. (1996). Measurement of lumbar lordosis: Evaluation of intraobserver, interobserver, and technique variability. Spine.

[55] Carman D.L., Browne R.H., Birch J.G. (1990). Measurement of scoliosis and kyphosis radiographs. Intraobserver and interobserver variation. J. Bone Jt. Surg..

[56] Lechner R., Putzer D., Dammerer D., Liebensteiner M., Bach C., Thaler M. (2017). Comparison of two-and three-dimensional measurement of the Cobb angle in scoliosis. Int. Orthop..

[57] Cracknell J., Lawson D.M., Taylor J.A. (2015). Intra-and inter-observer reliability of the Cobb measurement by chiropractic interns using digital evaluation methods. J. Can. Chiropr. Assoc..

[58] Dai J., Li Y., He K., Sun J. (2016). R-FCN: Object detection via region-based fully convolutional networks. Adv. Neural Inf. Process. Syst..

[59] Zhang S., Wen L., Bian X., Lei Z., Li S.Z. Single-shot refinement neural network for object detection. Proceedings of the IEEE Conference on Computer Vision and Pattern Recognition.

[60] Tsung-Yi L., Goyal P., Girshick R., He K., Dollar P. Focal loss for dense object detection. Proceedings of the IEEE International Conference on Computer Vision.

[61] Redmon J., Farhadi A. (2018). Yolov3: An incremental improvement. arXiv.

[62] Yi-Lang C. (1999). Vertebral centroid measurement of lumbar lordosis compared with the Cobb technique. Spine.

[63] Yang B.P., Yang C.W., Ondra S.L. (2007). A novel mathematical model of the sagittal spine. Spine.

[64] Suri A., Jones B.C., Ng G., Anabaraonye N., Beyrer P., Domi A., Choi G., Tang S., Terry A., Leichner T. (2021). Vertebral Deformity Measurements at MRI, CT, and Radiography Using Deep Learning. Radiol. Artif. Intell..

[65] Cho B.H., Kaji D., Cheung Z.B., Ye I.B., Tang R., Ahn A., Carrillo O., Schwartz J.T., Valliani A.A., Oermann E.K. (2020). Automated measurement of lumbar lordosis on radiographs using machine learning and computer vision. Glob. Spine J..

[66] Wang J., Zhang J., Xu R., Chen T.G., Zhou K.S., Zhang H.H. (2018). Measurement of scoliosis Cobb angle by end vertebra tilt angle method. J. Orthop. Surg. Res..

